# A toolbox for a structured risk-based prehabilitation program in major surgical oncology

**DOI:** 10.3389/fsurg.2023.1186971

**Published:** 2023-06-26

**Authors:** Svenja Sliwinski, Elisabeth Werneburg, Sara Fatima Faqar-Uz-Zaman, Charlotte Detemble, Julia Dreilich, Lisa Mohr, Dora Zmuc, Katharina Beyer, Wolf O. Bechstein, Florian Herrle, Patrizia Malkomes, Christoph Reissfelder, Joerg P. Ritz, Tim Vilz, Johannes Fleckenstein, Andreas A. Schnitzbauer

**Affiliations:** ^1^ Department of General, Visceral, Transplant and Thoracic Surgery, University Hospital Frankfurt, Goethe University Frankfurt/Main, Frankfurt/Main, Germany; ^2^ Institute of Sports Medicine, Goethe University Frankfurt/Main, Frankfurt/Main, Germany; ^3^ Department of General, Visceral and Vascular Surgery, Campus Benjamin Franklin, Charité-Universitätsmedizin Berlin, Berlin, Germany; ^4^ German Association for General and Visceral Surgery (DGAV), Surgical Work Force for Perioperative Medicine, Berlin, Germany; ^5^ Romed Klinik Prien am Chiemsee, Klinik für Allgemein- und Viszeralchirurgie, Prien am Chiemsee, Germany; ^6^ Department of Surgery, Universitätsmedizin Mannheim, Medical Faculty Mannheim, University of Heidelberg, Mannheim, Germany; ^7^ Helios Clinics Schwerin, Department for General and Visceral Surgery, Schwerin, Germany; ^8^ Department of General, Visceral, Thoracic, and Vascular Surgery, University Hospital Bonn, Bonn, Germany; ^9^ Department of Pain Medicine, Hospital Landsberg am Lech, Landsberg am Lech, Germany

**Keywords:** surgical oncology, prehabilitation, morbidity, risk assessment—methods, exercising, nutrition, mental wellbeing, PBM

## Abstract

Prehabilitation is a multimodal concept to improve functional capability prior to surgery, so that the patients’ resilience is strengthened to withstand any peri- and postoperative comorbidity. It covers physical activities, nutrition, and psychosocial wellbeing. The literature is heterogeneous in outcomes and definitions. In this scoping review, class 1 and 2 evidence was included to identify seven main aspects of prehabilitation for the treatment pathway: (i) risk assessment, (ii) FITT (frequency, interventions, time, type of exercise) principles of prehabilitation exercise, (iii) outcome measures, (iv) nutrition, (v) patient blood management, (vi) mental wellbeing, and (vii) economic potential. Recommendations include the risk of tumor progression due to delay of surgery. Patients undergoing prehabilitation should perceive risk assessment by structured, quantifiable, and validated tools like Risk Analysis Index, Charlson Comorbidity Index (CCI), American Society of Anesthesiology Score, or Eastern Co-operative Oncology Group scoring. Assessments should be repeated to quantify its effects. The most common types of exercise include breathing exercises and moderate- to high-intensity interval protocols. The program should have a duration of 3–6 weeks with 3–4 exercises per week that take 30–60 min. The 6-Minute Walking Testing is a valid and resource-saving tool to assess changes in aerobic capacity. Long-term assessment should include standardized outcome measurements (overall survival, 90-day survival, Dindo–Clavien/CCI®) to monitor the potential of up to 50% less morbidity. Finally, individual cost-revenue assessment can help assess health economics, confirming the hypothetic saving of $8 for treatment for $1 spent for prehabilitation. These recommendations should serve as a toolbox to generate hypotheses, discussion, and systematic approaches to develop clinical prehabilitation standards.

## Introduction

Major surgeries are one of the top three reasons of deaths in hospitals (1). The World Health Organization analyzed that $1 out of $7 is spent for the treatment of complications in hospitals worldwide (2, 3). Patients undergoing major surgeries have an increased risk of experiencing minor and major complications, which may impact quality of life and lead to short-term failure to rescue and increased mortality (4, 5). Data on the prevalence of such surgical complications range between 15% and 50%, which covers uncertainty unless systematically assessed by the current gold standard, which is the Dindo–Clavien classification of surgical complications or its evolution to the comprehensive complication index (6–10). Taking these scientific aspects into account, major surgery may be considered one of the greatest contributors of clinical deaths despite its lifesaving and curative role being an early therapeutic option in most solid cancers. As the Western societies are getting older, and the average age to get diagnosed with a malignant tumor is currently 65, it is crucial to address a clear strategy to identify and improve modifiable factors before surgery and thus mitigate the risk of experiencing an adverse course after major surgery (11).

Clinical treatment strategies have been constantly improved over the last 20 years to increase the safety and quality surgical treatments have been implementing, e.g., the use of checklists, interdisciplinary board decision-making, treatment at specialized centers, a refinement of neoadjuvant and adjuvant treatment strategies, enhanced recovery after surgery, and minimally invasive techniques (2, 12, 13). One field that is gaining importance and popularity recently is prehabilitation. This means the strategy of adequately preparing the patient for a surgical procedure by identifying and improving modifiable factors in advance of the surgical procedure. In general, prehabilitation is based on three pillars that are physical activity, healthy nutrition, and psychosocial wellbeing. Patient–blood management, a bundle strategy to correct anemia before surgeries, should also be mentioned in this context and contributes to better outcomes after oncologic surgeries (14–17).

To date, there is debate among healthcare professionals as to which systematic and structured assessments are most appropriate for patients to assess their individual risk profile, what kind of prehabilitation modalities should be used, and how long patients need to exercise to see an effect without increasing the risk for progression of the underlying disease and thus worsening prognosis from a potentially beneficial intervention.

Strikingly, prehabilitation is only marginally implemented in clinical settings and still lacks reimbursement or awareness by the stakeholders. The required setup in a hospital is cost intensive as, e.g., dedicated and mostly non-existent personnel are required. The evaluation of cost-effectiveness is the subject of current research, but first theoretical algorithms estimate a small return on investment. In addition, approaches are faced with individual barriers, e.g., patients need to be motivated to come to hospitals/gyms for multiple appointments prior to surgery (18).

In summary, the current overview of studies and recommendations suggests a variety of approaches due to the heterogeneity of provided data. Yet, there is no general recommendation (“toolbox”) mapping the best of prehabilitation concepts. The aim of this review is to systematically screen current literature to identify the best available evidence to obtain structured and useful assessment tools to measure patient risk before surgeries and prehabilitation. In addition, we aim to identify the most promising interventions for the single elements of prehabilitation in addition to the best dose relationships, i.e., the duration of exercise interventions, all this considering the risk–benefit of delaying oncologic surgeries and identifying strategies for a reasonable and broad penetration and traction of measurable prehabilitation in clinical or remote settings. The targeted groups of interest are all adult surgical oncologic indications in abdominal, thoracic, urologic, and gynecologic surgeries.

## Methods

The systematic scoping review was developed using guidance from the Preferred Reporting Items for Systematic Reviews and Meta-Analysis (PRISMA) guidelines (19).

### Search strategy

The electronic databases PubMed, Google Scholar, and AWMF Leitlinienportal (Official German Medical Guidelines) were systematically queried to find applicable articles published between the years 1991 and 2021. The search was structured in three relevant blocks using the following terms, PICO-searches and MeSH terms where applicable: risk assessment (RAI-C scoring, Charlson Comorbidity Index, Patient Blood Management, QLQ-C-30 questionnaire, Timed-Up and Go Testing, 6 Minute walking test, Dindo-Clavien scoring, 90-day surgical mortality (specific search after deciding for the tools that will be used from clinical use), prehabilitation (PICO search: P(surgery) I(prehabilitation) O(survival) to obtain relevant results; (((((((surgery) AND (prehabilitation)) AND (complications)) AND (survival)) NOT (cardiac surgery)) NOT (orthopedic surgery)) NOT (emergency). MESH: (((((“surgery”[MeSH Subheading] OR “surgery”[All Fields] OR “surgical procedures, operative”[MeSH Terms] OR (“surgical”[All Fields] AND “procedures”[All Fields] AND “operative”[All Fields]) OR “operative surgical procedures”[All Fields] OR “general surgery”[MeSH Terms] OR (“general”[All Fields] AND “surgery”[All Fields]) OR “general surgery”[All Fields] OR “surgery s”[All Fields] OR “surgerys”[All Fields] OR “surgeries”[All Fields]) AND (“prehabilitative”[All Fields] OR “preoperative exercise”[MeSH Terms] OR (“preoperative”[All Fields] AND “exercise”[All Fields]) OR “preoperative exercise”[All Fields] OR “prehabilitation”[All Fields]) AND (“complicances”[All Fields] OR “complicate”[All Fields] OR “complicated”[All Fields] OR “complicates”[All Fields] OR “complicating”[All Fields] OR “complication”[All Fields] OR “complication s”[All Fields] OR “complications”[MeSH Subheading] OR “complications”[All Fields]) AND (“mortality”[MeSH Subheading] OR “mortality”[All Fields] OR “survival”[All Fields] OR “survival”[MeSH Terms] OR “survivability”[All Fields] OR “survivable”[All Fields] OR “survivals”[All Fields] OR “survive”[All Fields] OR “survived”[All Fields] OR “survives”[All Fields] OR “surviving”[All Fields])) NOT (“thoracic surgery”[MeSH Terms] OR (“thoracic”[All Fields] AND “surgery”[All Fields]) OR “thoracic surgery”[All Fields] OR (“cardiac”[All Fields] AND “surgery”[All Fields]) OR “cardiac surgery”[All Fields] OR “cardiac surgical procedures”[MeSH Terms] OR (“cardiac”[All Fields] AND “surgical”[All Fields] AND “procedures”[All Fields]) OR “cardiac surgical procedures”[All Fields] OR (“cardiac”[All Fields] AND “surgery”[All Fields]))) NOT (“orthopaedic surgery”[All Fields] OR “orthopedics”[MeSH Terms] OR “orthopedics”[All Fields] OR (“orthopedic”[All Fields] AND “surgery”[All Fields]) OR “orthopedic surgery”[All Fields])) NOT (“emerge”[All Fields] OR “emerged”[All Fields] OR “emergence”[All Fields] OR “emergences”[All Fields] OR “emergencies”[MeSH Terms] OR “emergencies”[All Fields] OR “emergency”[All Fields] OR “emergent”[All Fields] OR “emergently”[All Fields] OR “emergents”[All Fields] OR “emerges”[All Fields] OR “emerging”[All Fields])) AND (y_5[Filter])), nutrition (Surgery and immunonutrition, AWMF screening, PICO search: P(surgery) I(immunonutrition) O(survival)), delay of surgery (delaying cancer surgery and mortality). Inclusion criteria were clinical trials that published data on prehabilitation in adult oncologic surgery of the abdomen, thoracic, gynecologic oncologic surgery, and urology. Risk assessment data had to include outcome measurement (survival and complication rates). Nutrition data should comprise data from clinical trials, randomized controlled trials, or systematic reviews and meta-analyses. Delaying surgery focused on oncologic treatments as outlined for prehabilitation above. Exclusion criteria were indications other than those mentioned above, pediatric surgeries, cardiovascular surgeries, trauma surgeries, missing data on exercising and assessment modalities, and missing outcome data.

### Study selection and data extraction

Studies were included when they analyzed risk assessment scores and risk factors. They were included when they used either exercise tests or nutritional therapy, or psychoeducation as a prehabilitation modality and assessed the QoL (Quality of life), mortality, costs, or length of hospital stay postoperatively. Studies for which the full text was not available were excluded, as were studies of patients undergoing orthopedic, pediatric, trauma, and cardiac surgery and opinion, statement, position papers, letters to the editor, guidelines, symposium protocols, study protocols, advisory reports, manuals, commentaries, or recommendation papers. Case reports, opinion papers, animal studies, and studies other than the English language were also excluded. After the removal of double hits from the search results, three reviewers (EW, SS, and AAS) independently screened and selected potentially eligible studies. After consensus was reached in this initial selection procedure, the reviewers independently reviewed the full text of the selected studies to determine the final suitability for inclusion based on the established inclusion criteria. To include additional relevant studies, after full-text assessment, the references sections of papers were screened, and relevant papers were chosen based on the above-described criteria.

### Risk of bias

Risk of bias is regarded high due to the high number of lower level of evidence, especially large cohorts (20).

### Recommendations

Based on the evidence found in the literature, statements were derived from the findings. The GRADE (Grading of Recommendations Assessment, Development, and Evaluation) for the clinical guidelines pathway was followed, which is shown in Table 1 (21).

**Table 1:** Recommendations and suggestions for use, further exploration, and generation of hypotheses to strengthen prehabilitation as a standard of care and generate the political will to reimburse the medical intervention.

Category	Recommendation/suggestion
Risk assessment and safety considerations	*#1: To generate comparable and measurable baseline data of patient risk, the standard patient history should be complemented by at least two different risk assessments like the Risk Assessment Index, the Charlson Comorbidity Score, the ASA or ECOG score, **and** the measurement of sarcopenia and/or the assessment of the 6-MWT or the TUG. This is suggested to be implemented into the structured patient pathway to surgery in every setting to avoid double-documentation and mitigate waste of time.*
*Exercise recommendations: type*, duration, frequency	#2: Unfit patients should try to increase their exercise capacity to at least 75 min of vigorous (conversation is difficult but breathing fast) or 150 min of moderate (conversation possible, breathing increased) intensity per week. Sedentary time should be reduced, and stabilization and resistance training should be done at least 2 times per week.
	#3: A specific exercise program before surgical procedures is suggested to be performed for at least 3–6 weeks and might consist of 3–4 times per week moderate aerobic interval training when performed remotely, and moderate to vigorous training when performed in a completely supervised setting. The sessions should last between 30 and 60 min. Patient progress should be monitored or supervised with adequate measures and safety interventions, especially when done remotely.
Exercise testing	#4: The 6-MWT is suggested to be performed as a baseline and post-prehabilitation exercise testing tool in a clinical setting. A CPET might be considered in case that the infrastructure is easily available. The Karvonen method is suggested to calculate the individual program that might be adjusted considering additional individual risk factors like heart rate modulating drugs.
	#5: The extension of an exercise program beyond 4 weeks in patients undergoing neoadjuvant or bridging therapies to the operation might be considered. Patients not undergoing these kinds of strategies should be operated after 4 weeks of preparation at the latest.
Outcome measures	#6: Patients require a baseline risk assessment including exercise testing and a preoperative/post-prehab assessment to measure improvement or deterioration. This might include real-time exercise measurements as well as patient-reported outcomes.
	#7: Surgical outcomes should be measured in a structured way. The following outcome parameters should be considered: diagnosis (ICD), procedure (OPS-coded), complication assessment with the Dindo–Clavien score or the comprehensive complication index®, and 90-day overall survival. Long-term follow-up, impact on oncologic outcomes per indication, as well as in-depth analysis of the individual complications are suggested.
Nutrition	#8: Patients should be screened with the standardized nutritional risk surveys on the patient pathway either directly or as a (digitized) self-reporting tool. Based on the results, professional nutritional consulting should be performed. Protein-enriched (immune) nutrition might be generally considered while a patient is in the prehabilitation program.
Patient–blood management	#9: All patients in prehabilitation programs should undergo a structured patient blood management pathway, and anemic patients should receive special focus and attempts to correct the anemia.
Mental wellbeing	#10: Patients in prehabilitation programs should undergo a quality-of-life assessment before and after the program to measure improvement or deterioration. Every patient should be asked whether they want psycho-oncologic counseling. Stress reducing and motivational behavior strategies might increase general compliance, motivation, and surgical success.
Economic potential	#11: Key performance indicators for the economic success of a prehabilitation program should include an individual and detailed complete cost-revenue calculation for each patient including stays in normal wards, intermediate care wards, and ICUs, and readmission. Long-term costs might be considered by payers to measure the effects on oncologic success. Payers should analyze the potential of establishing reimbursement codes to implement prehabilitation as a refundable medical service.

ASA, American Society of Anesthesiology Score; ECOG, Eastern Co-operative Oncology Group; 6-MWT, 6-Minute Walking Testing; TUG, Timed Up and Go Testing; CPET, cardiopulmonary exercise testing; ICUs, intensive care units; ICD, international statistical classification of diseases and related health problems; OPS, operations and procedures key (Schluessel).

## Results

### Literature search report and PRISMA flow chart for selection of studies

**Figure 1:**
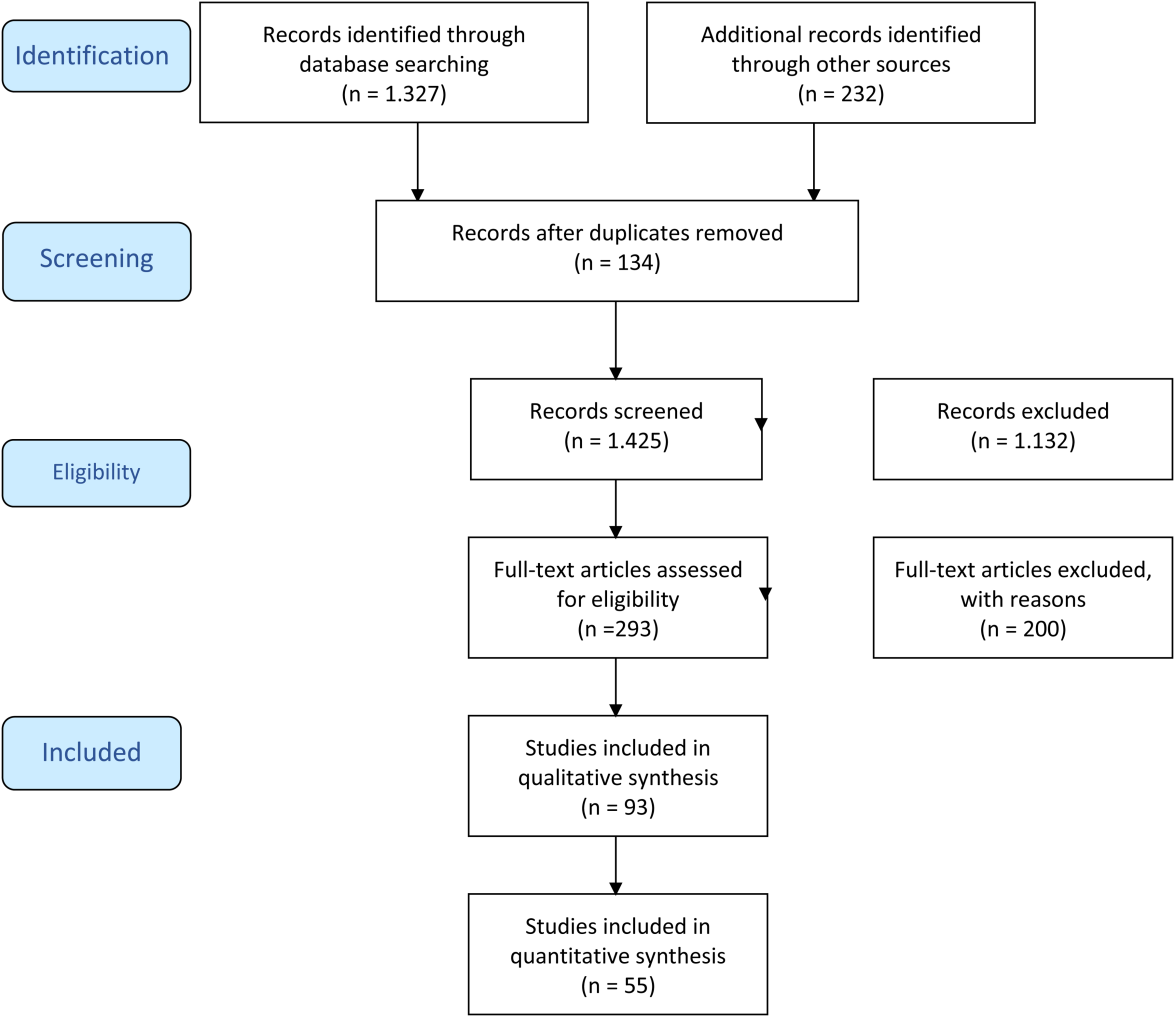
PRISMA flowchart of selection of studies.

The literature search report identified 1,559 manuscripts of which 134 were doubles; 1,132 were not suitable after screening the titles and abstracts and another 195 were not suitable because of the defined criteria outlined above. The selection process is outlined in the PRISMA flowchart in Figure 1 (19). A total of 93 studies were identified to be suitable for the review.

### Description of the level of evidence and bias

The Oxford levels for evidence-based medicine were applied to classify the quality of studies identified. From 93 identified studies, 13 were defined as class 1 evidence delivering solid meta-analysis of high-quality trials; 41 trials were defined as class 2 delivering data from randomized controlled trials or large cohorts with a dramatic effect; 23 trials were class 3 consisting of a cohort and observational studies; 15 were class 4 cohort, observational, and case (control) series with low patient numbers; and finally, 1 publication was classified as class 5 evidence as it depicted a standpoint about the topic. Bias was high in the screened trials, as most trials were simply not randomized, blinded, or dropouts, and withdrawals were not described adequately.

Finally, only class 1 and 2 articles with one selected class 3 evidence article (in total *n* = 55) (class 3 selected because of high-quality economic work-up) were considered for a deeper analysis and as a valid source to build recommendations and suggestions about the investigated topics. Notably, one article could cover several of the following topics so the following numbers don’t add up to the total.

The topics covered by this review were “risk assessment tools” (*k* = 13; *n* = 3,686,465), “prehabilitation, exercise testing, and physical activity” (*k* = 23; *n* = 352,898), “delay of surgery and risk of oncologic progression” (*k* = 10; *n* = 1,846,995), “nutrition” (*k* = 20; *n* = 565,843), “patient blood management” (*k* = 2; *n* = 3,008), mental wellbeing (*k* = 10; *n* = 8,100), and “economics and prehabilitation” (*k* = 4; *n* = 290,522). A closer description can be found in Table 2.

**Table 2:** Studies included in final recommendation/suggestion generating synthesis analysis.

Ref.	Year	Population	Type	Oxford levels of evidence	Sample size (*n*)	Age (years)	Focus
ACSM Guidelines (22)	2018	General	Guideline	2	n.a.	n.a.	Prehab, mental
Arends et al. (23)	2017	Surgical oncology	Guideline	2	n.a.	n.a.	Nutrition
Arya et al. (24)	2020	Major surgery	Cohort study	2	1,879,372	60.7 (13.1)	Assessment
ATS-Statement (25)	2002	General	Guideline	2	n.a.	n.a.	Assessment
Bagaria et al. (26)	2019	Surgical oncology	Cohort study	2	4,685	71	Delay time to surgery
Barberan-Garcia et al. (27)	2018	GI surgery	Randomized controlled trial	2	125	71	Prehab, nutrition, PBM, mental
Barberan-Garcia et al. (28)	2019	GI surgery	Randomized controlled trial	2	125	71	Economy
Bhatia and Kayser (29)	2019	Thoracic surgery	Randomized controlled trial	2	151	64	Prehabilitation
Boden et al. (30)	2018	Upper GI surgery	Randomized controlled trial	2	432	I: 63.4 C: 67.5	Prehabilitation
Boden et al. (31)	2020	Upper GI surgery	Randomized controlled trial	2	432	I: 63.4 C: 67.5	Economy
Bolshinsky et al. (32)	2018	GI surgery	Systematic review	2	2,883	n.a.	Prehab, nutrition, PBM
Bourgade et al. (33)	2014	Surgical oncology	Systematic review	2	n.a.	n.a.	Delay time to surgery
Briggs et al. (34)	2022	Surgical oncology	Systematic review	2	513	61.2–72	Prehab, nutrition, mental
Bruns et al. (35)	2018	GI surgery	Meta-analysis	1	583	63	Nutrition
Cavalheri et al. (36)	2017	Thoracic surgery	Meta-analysis	1	167	54–72.5	Prehabilitation
Ekblom-Bak et al. (37)	2019	General	Cohort study	2	266,109	18–74	Prehab, nutrition
Ekblom-Bak et al. (38)	2020	General	Cohort study	2	64,970	18–75	Prehab, nutrition
Ekblom-Bak et al. (39)	2021	General	Cohort study	2	3,693	60	Prehab, nutrition
Elit et al. (40)	2013	Surgical oncology	Cohort study	2	9,417	n.a.	Delay time to surgery
Figueiredo et al. (41)	2018	Surgical oncology	Cohort study	2	470	66	Delay time to surgery
Fuertes-Guiró and Viteri Velasco (42)	2020	Surgery	Meta-analysis	1	289,176	n.a.	Assessment economy
Fulop et al. (43)	2021	GI surgery	Randomized controlled trial	2	149	70	Prehab, nutrition, mental
Giannitsi et al. (44)	2019	Heart failure	Systematic review	2	3,880	n.a.	Assessment
Hall et al. (45)	2017	Surgery	Cohort study	2	6,856	60.7	Assessment
Hanna et al. (46)	2020	Oncology	Meta-analysis	1	1,272,681	n.a.	Delay time to surgery
Howard et al. (47)	2019	Major surgery	Cohort Study	3	116	59	Economy
Lambert et al. (48)	2021	Surgical oncology	Meta-analysis	1	1,955	n.a.	Prehabilitation
Lau and Chamberlain (49)	2020	Surgical oncology	Meta-analysis	1	929	n.a.	Prehab, nutrition, mental
Levett et al. (50)	2018	General surgery	Guideline	2	n.a.	n.a.	Assessment
Liu et al. (51)	2022	Surgical oncology	Meta-analysis	1	1,553	n.a.	Prehab, nutrition, mental
Looijard et al. (52)	2018	Surgical oncology	Systematic review	2	496	64.5–71.1	Prehab, nutrition
McKenna et al. (53)	2020	Surgical oncology	Cohort study	2	205,840	66	Nutrition
Meng et al. (54)	2018	Surgical oncology	Systematic review	2	1.516	67.2	Assessment
Minnella et al. (55)	2016	GI surgery	Meta-analysis	1	105	55–88	Prehab, nutrition, mental
Minnella et al. (56)	2021	Surgical oncology	Randomized controlled trial	2	70	I: 69.7 C: 66.0	Prehab, nutrition, mental
Mirkin et al. (57)	2018	Surgical oncology	Cohort study	2	14,807	n.a.	Delay time to surgery
Moran et al. (58)	2016	General surgery	Meta-analysis	1	435	34.8–71.3	Prehabilitation
Palumbo et al. (59)	2020	Surgical oncology	Cohort study	2	23,967	70	Assessment
Piraux et al. (60)	2021	Surgical oncology	Systematic review	2	645	n.a.	Prehabilitation
Polverini et al. (61)	2016	Surgical oncology	Cohort study	2	420,792	59.4	Delay time to surgery
Probst et al. (62)	2017	Major surgery	Meta-analysis	1	7,116	n.a.	Nutrition
Shah et al. (63)	2018	General surgery	Cohort study	2	984,550	58.2	Assessment
Shah et al. (64)	2020	Minor surgeries	Cohort study	2	28,059	56.7	Assessment
Sheill et al. (65)	2020	Surgical oncology	Systematic review	2	1,735	n.a.	Prehabilitation
Shinall et al. (5)	2020	General surgery	Cohort study	2	432,828	61	Assessment
Simunovic et al. (66)	2009	Surgical oncology	Cohort study	2	7,989	n.a.	Delay time to surgery
Strohl et al. (67)	2016	Surgical oncology	Cohort study	2	112,041	61.8	Delay time to surgery
Tew et al. (68)	2018	Major surgery	Guideline	2	1,057	n.a.	Prehabilitation
Thillainadesan et al. (69)	2020	General surgery	Meta-analysis	1	3,026	65–81	Prehab, nutrition, mental
van Kooten et al. (70)	2021	GI surgery	Systematic review	2	n.a.	n.a.	Assessment
Varley et al. (71)	2020	General surgery	Cohort study	2	36,261	57.5	Assessment
Waterland et al. (72)	2021	Surgical oncology	Meta-analysis	1	1,700	55–84	Prehab, nutrition, mental
Weimann et al. (73)	2014	General surgery	Guideline	2	n.a.	n.a.	Nutrition
Xu et al. (74)	2019	Surgical oncology	Cohort study	2	12,102	62.5	Delay time to surgery
Yu et al. (75)	2020	Surgical oncology	Meta-analysis	1	5,983	n.a.	Nutrition

GI, gastrointestinal.

Topics cover Risk assessment, Prehabilitation, Nutrition, Delay time to surgery, Definition of major surgery, and Economic potential of prehabilitation.

#### Risk assessment tools

We identified activity of daily living (ADL), Age, Risk Analysis Index (RAI score), Charlson Comorbidity Index (CCI), Clinical frailty scale (CSHA), Sarcopenia, (modified) frailty index (mFI), American Society of Anesthesiology Score (ASA), Timed Up and Go Testing (TUG), 6-Minute Walking Testing (6-MWT), Eastern Co-operative Oncology Group (ECOG), and Psoas muscle Index being the most frequently used risk assessment tools in the published literature. There is no general international agreement or highly evident recommendation, on which scores should be used to identify the individual risk profile of (major) surgical patients. Thirteen of the 26 analyses have an Oxford level of evidence of 2 or better and accumulate almost 3.7 Mio. patients analyzed (5, 24, 25, 42, 44, 45, 50, 54, 59, 63, 64, 70, 71). Six include the ADL, 10 evaluate age as a risk factor, 5 studies highlight the RAI score, 4 picked up the CCI, 5 used ASA, 4 assessed the 6-MWT, and 2 measured sarcopenia by muscle density in imaging (multiple answers).

The RAI score consists of age, clinical risk factors, and a modified ADL checklist that generates a scoring that was validated in multimillion patients in the United States. It has shown a highly significant association between the risk groups in various studies and is an excellent marker for frailty. Moreover, it was validated as a highly correlative marker for failure to rescue after major surgical procedures with rising scores (5, 24, 45, 63, 64). Similar results were obtained by the ASA and the CCI score, which, however, do consider fewer variables than the RAI score that can be assessed within 2–5 min and delivers a highly valuable clinical assessment.

#### Exercise training recommendations in prehabilitation: type, duration, frequency, and intensity

The literature research revealed 24 out of 55 studies with Oxford evidence levels of 1 and 2 focusing on prehabilitation prior to intra-abdominal or thoracic surgery predominantly in the oncologic area and including 353,014 patients for analysis (27, 29–32, 34, 36–39, 43, 48, 49, 51, 52, 55, 56, 58, 60, 65, 68, 69, 72). One guideline was related to the general population with recommendations for physical activity (22).

### Type of exercise

A total of 18 (78%) studies investigated different types of exercise interventions and exercise testing. Thirteen studies applied inspiratory muscle training (IMT) to prepare patients for surgery (30, 32, 36, 43, 48, 49, 52, 55, 58, 60, 68, 69, 72). Five studies reported high intense interval training (HIIT) as modality (27, 29, 34, 60, 68), and in five studies, the intensity was controlled with various target measurements of intensity (27, 29, 55, 56, 72). Two interventions were performed as purely home based (55, 68); all other trials were carried out as hybrid trials, and only five trials offered individualized exercising programs tailored to each patient (27, 29, 49, 56, 68). Most trials were supervised for at least the first session or for the HIIT exercises by a physician, physiotherapist, or other qualified medical staff.

### Duration, frequency, and intensity of exercise

Frequency and duration differed between the evaluated clinical trials. There was a range between 3 times per day to 5 times per week using the time period of 1–6 weeks before the surgical procedure. The individual session lasted between 20 and 75 min. The most popular load was moderate- to high-intensity interval training [common protocols: (i) 2 min high intensity vs. 3 min low intensity, (ii) 15 s of very high intensity vs. 15 s of passive rest for 4 min and additional 4 min rest, or (iii) continuous moderate]. Patients were instructed personally or with leaflets. In most studies, the patients received a standard of care control group intervention explaining the benefits of breathing or recommending some low-intensity exercise before surgery.

### Exercise testing

A variety of methods to assess aerobic capacity and endurance following prehabilitation programs has been described among the 23 studies: 6-MWT (*k* = 17) (22, 25, 27, 29, 32, 34, 36, 43, 44, 48, 49, 51, 55, 56, 58, 68, 72). Cardiopulmonary exercise testing (CPET) analyzed the VO_2max_ or Metabolic Equivalents of Task (*k* = 9) (22, 27, 29, 32, 34, 44, 50, 68, 72), patient-reported outcomes [PROMS, i.e., perceived exertion with the Borg scales or other comparable scales like the Visual Analog Scale (VAS) or Numeric Rate Scale (NRS); *k* = 7] (22, 25, 29, 34, 55, 56, 72), a combination of vital signs (e.g., HR, the BP, or both; *k* = 7) (22, 27, 29, 36, 43, 50), or the Forced 1 Sec. Expiratory Volume (FEV1, *k* = 6) (22, 27, 29, 36, 43, 50). Other tests such as muscle strength, oxygen saturation, or Diffusion Capacity of the Lungs for Carbon Monoxide (DLCO) played a minor role (22, 25, 27, 29, 34, 47, 60).

#### Impact of prehabilitation on tumor growth

To better understand the duration of prehabilitation modalities, especially in surgical oncology, the data on tumor progression before surgical procedures have been analyzed. Ten studies were identified as grade 1 or 2 Oxford level of evidence classified. Those studies evaluated most solid cancers in the thorax and abdomen and found that a delay of surgery for 30 days is not associated with adverse outcomes in surgical patients (26, 33, 40, 41, 57, 61, 66, 67, 74). However, a meta-analysis by Hanna et al. revealed an increased risk for additional tumor-associated deaths by 6%–8% for every 4 weeks of every oncologic treatment delay, which must be weighed against the potential benefits of prehabilitation and its morbidity-reducing effects (46).

##### Outcome measures

Outcome measurements were heterogeneous and clinically not necessarily meaningful. For a clinically relevant outcome measure, it is important to be easily implemented in the clinical workflow and pathway. Overall, there is a general agreement that good cardiopulmonary fitness is associated with a decreased risk for cardiovascular risk in the general population (37). Patients with heart failure have lower functional capacity and should be assessed routinely with ergometer-based methods (maximal exercise test) prior to exercise interventions. From a pragmatic point of view, ergometer-based assessment is often replaced by 6-MWT and has been used in 10 out of 19 studies reporting outcome measures as well as 23 out of 37 studies investigating exercise testing. This decision is based on strong to moderate correlations between methods (44) and can thus be transferred into the surgical setting, where a lower anaerobic threshold is associated with a higher risk for 90-day mortality after e.g., esophagectomies (65). We identified four RCTs included in this analysis that revealed significant improvements for perioperative complications: The studies showed complications to be reduced by 51% [relative risk (RR): 0.51; 95% CI: 0.3–0.8; *p* = 0.001], including shorter ICU and hospital stays, lower rates of hospital readmission rates (drop from 18% to 3%, *p* = 0,009), and delivering high compliance (up to 87%). Other studies revealed pulmonary complications to be reduced by 50% (HR 0.48; 95% CI: 0.30–0.75; *p* = 0.001), or functional capacity to be improved by 130% [interquartile range (IQR): 112–137; *p* < 0.001], as assessed by 6-MWTs (27, 29, 30, 43).

Importantly, there were only single reports about intervention-related adverse events in predominantly patients who were older than 60 years, which displays strong safety for the patients in a high-intensity interval training advocating for a patient-empowering home-based setting with an unsupervised moderate to vigorous interval training in case of exclusion of major cardiopulmonary risk factors. Endpoint measurements are heterogeneous and should include measurable and meaningful clinical endpoints for short-term outcome quality and long-term oncologic outcome stratified to the underlying disease.

#### Nutrition items

Nutritional assessment tools are important to identify patients with an impaired nutritional status and support those that require medically indicated nutritional supplementation. This is important as it is known that an impaired nutritional status may be associated with increased complication rates like surgical site infections.

A total of 20 articles with an Oxford level of evidence of 2 or better analyzed nutritional recommendations before surgical procedures (23, 27, 32, 34, 35, 37–39, 43, 49, 51–53, 55, 56, 62, 69, 72, 73, 75), of which 12 used a nutritional assessment tool or self-reporting to measure the nutritional status of an individual patient (22, 27, 35, 37–39, 43, 53, 56, 58, 69, 72, 73). Fourteen studies recommended a specific nutrition support mode that consisted predominantly of specific protein supplementation varying between the publication and/or the regular supplementation with immunonutrition, ranging between 3 days and 6 weeks before surgery. Compliance ranged between 72% and 100%. Outcome measures like the length of stay or the occurrence of surgical site infections could be reduced in some trials. Generally, most authors are advocating for a protein-enriched (immune-) nutritional protocol before major surgery, focusing on mitigating the risk of sarcopenia, post-aggressive metabolism, and malnutrition.

#### Patient blood management items

Anemia is associated with adverse outcomes after surgical procedures. In more than 95% of all cases, iron deficiency is the leading reason for anemia and those can be corrected with intravenous application of iron. Indeed, several studies have shown that the oncologic outcomes of patients who are not anemic are better than the outcomes of those who are. However, there are contradicting data from purely iron supplementing clinical trials as well (76). In contrast, the structured implementation of patient blood management (PBM) was shown to reduce the requirements for transfusions, i.e., the ratio of anemic patients undergoing operations, and was associated with better oncologic outcomes in multiple real-life cohorts and scenarios (14–17). Only two high-quality studies in abdominal surgery identified anemic patients and tried to correct the anemia with i.v. iron injections in accordance with the recommendations of the patient blood management associations (14, 15, 27, 32).

#### Mental wellbeing

Mental (or psychosocial) wellbeing is key to success in any medical treatment. However, personalities, resilience, and coping mechanisms are as heterogeneous as patient risk factors. Psychosocial factors can be measured; stress and other adverse factors can be mitigated systematically, and thus may have a positive influence on the patients’ experience before and after a surgical intervention. Only 10 high-quality studies out of 55 hits considered mental wellbeing as an outcome measure in their program (22, 27, 34, 43, 49, 51, 55, 56, 69, 72). There was a heterogeneous mix of behavioral strategies to improve the quality of life, motivational interviews, psychological support, anxiety, stress-reducing approaches, and relaxation strategies as the main tools to improve or keep psychosocial wellbeing in patients. The most frequently used tools include the SF-36 (short-form 36), HADS (hospital anxiety and depression scale), and CGA (comprehensive geriatric assessment). Only 4 of the 10 studies evaluated the effect of the intervention and concluded that behavioral strategies can increase compliance to exercise (70%–90%) by increased motivation and significantly reduced anxiety in patients during their surgical experience (reduction in anxiety score, *p* = 0.03).

#### Economic potential measurement (cost-effectiveness)

Prehabilitation to date is not reimbursed by any payers in any healthcare system, to our knowledge. This means that surgeons, dedicated care nurses, and other healthcare professionals use extra time, extra effort, and extra money to improve patient outcomes. For the evaluation of the economic potential, all four studies with an Oxford level of evidence level III or higher were included in the evaluation (28, 31, 42, 47). There was a significant benefit of prehabilitation on postoperative complications reflected by the cost-efficiency of a preoperative intervention. Simply expressed, every 1$ that was invested in prehabilitation led to a saving of 8$ in the postoperative course, which is a tremendous return on investment (31). These findings were just confirmed by the group of Howard, having established a trimodal prehabilitation program at their hospital reducing minor and major complications, leading to an economic advantage of prehabilitated patients of $65,000 vs. emergency patients and of $25,000 vs. routine elective patients (47).

Based on the findings from the extractions, recommendations were made for application, further systematic research, and evaluation and are displayed in Table 1.

## Discussion

As surgeons and anesthesiologists, we do care about our patients and try to avoid harm. Surgery is among the top risks of hospital deaths after nonemergent operations (1). This requires careful selection and a risk–benefit assessment that weighs in existing and modifiable risk factors and the potential of failure to rescue after surgery. Prehabilitation has, therefore, been recognized as a potential game-changer not only for selecting but also for increasing the ratio of patients who do not experience preventable harm, which still accounts for 1 out of 3 adverse events that occur in a hospital (13). Data that promote the establishment of prehabilitation are promising and show that there is a high potential to reduce the number of complications by 50% and that there is a realistic chance to simultaneously save $8 for care after surgery for every $1 invested into prehabilitation and proper evaluation and preparation of the patient before a major surgical procedure (27, 31).

To our best knowledge, prehabilitation is not an established and reimbursed treatment in healthcare systems worldwide, and it is dependent on enthusiastic clinical champions that shape a better understanding of the field by generating class 1 and 2 evidence. The field, however, is thriving at this moment making it challenging to keep up with the new evidence that emerges every month. Prehabilitation should be embedded in the whole clinical pathway and needs to be established for the patient immediately and fast. The establishment of an individualized prehabilitation program requires the knowledge of measurable and comparable patient risk factors. In this review, multiple highly evident risk assessment tools were identified, which in combination deliver a meaningful, reproducible, and structured assessment and have been proven to be associated with outcomes.

**Figure 2:**
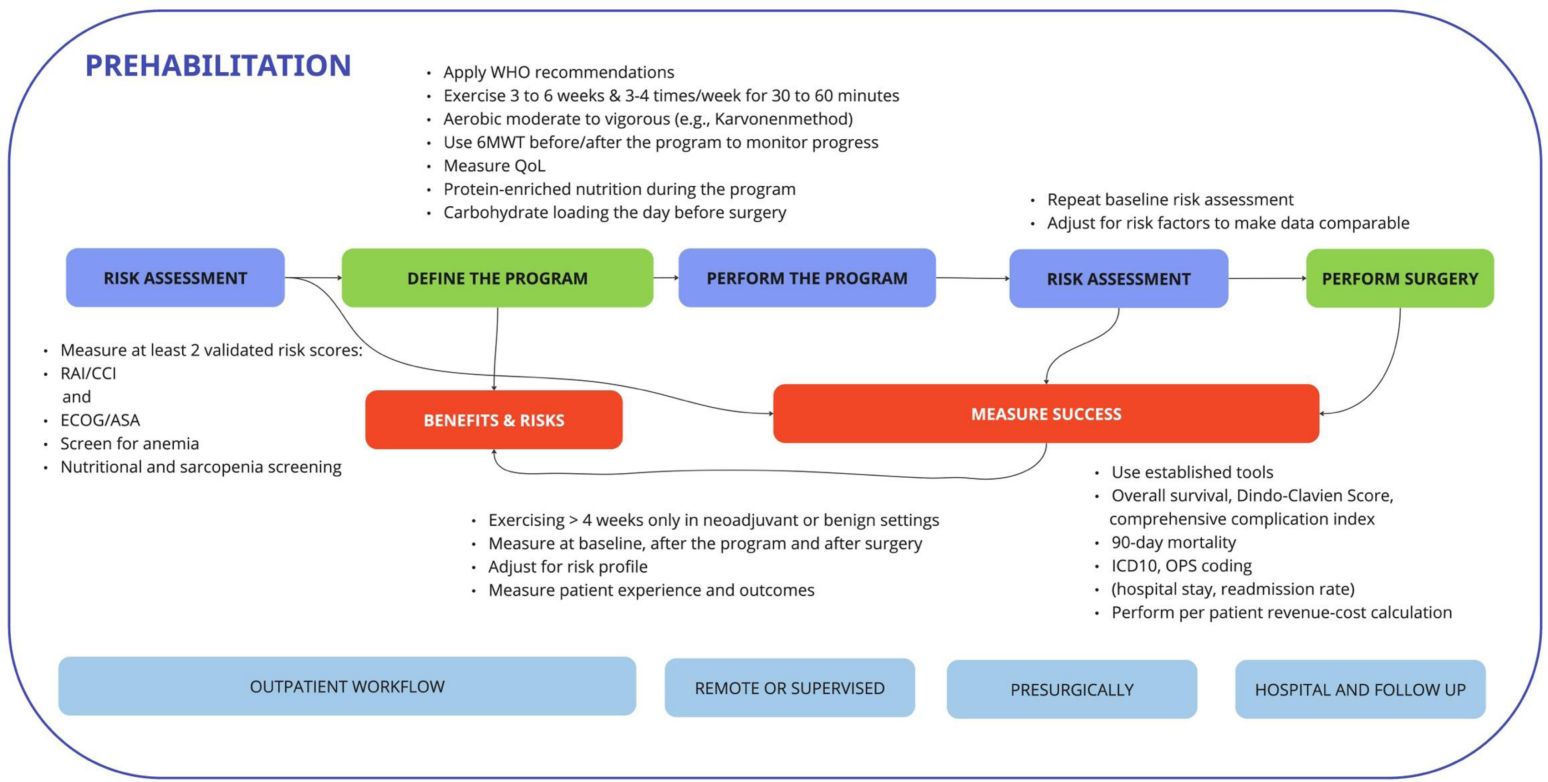
A workflow for the prehabilitation pathway that when implemented into routine procedures of the hospital workflow is likely to decrease the workload of surgeons, anesthesiologists, physiotherapists, and specialized nurses. Measurement of success based on baseline and reassessments as well as meaningful outcome measurements is key to success when implemented.

Exercise testing, the interventions of prehabilitation, and their duration remain the biggest challenges of definition in the literature. Multiple authors recommend dedicated pretraining exercise diagnostics to assess exercise capacity, likely comparable to professional sports. The type, frequency, duration, and intensity of each exercise are also the subject of discussion and ongoing research. However, real-life infrastructure to perform this kind of performance diagnostics in all patients is barely available considering the number of patients requiring surgical procedures. Most high-intensity exercises may appear too challenging, especially in view of the elderly. To date, prehabilitation has been shown to be effective when performed in clinical trials but is highly cost intensive, and lacks infrastructure as well as personnel in most hospitals. Patients, nonetheless, are motivated and compliance seems high with a proposed adherence ratio ranging from 70% to 100%. Therefore, based on our findings, we suggest a pragmatic approach (“toolbox”) for the clinical implementation of prehabilitation concepts (see also Figure 2).

The assessment of the risk factors with validated scores like the RAI score, the Charlson comorbidity index, the ASA score, or the ECOG score leads to highly reliable discrimination between patients (5, 54, 59, 63). Precise patient history is still critically important and needs to be regarded as the gold standard in patient and doctor interaction as Faqar-Uz-Zaman et al. have shown in a large double-blinded trial in patients with abdominal pain in an emergency room setting (77, 78). Although exercise testing such as CPET is the gold standard for assessing functional capacity, the 6-MWT can provide reliable information about the patient's daily activity and short-term prognosis, especially in patients with heart failure (HF) (chronic stable or acute decompensation). The 6-MWT is an easy-to-perform, widely available, and well-tolerated test for assessing the functional performance of patients with HF in daily clinical practice (44).

However, contraindications against the performance of a 6-MWT and/or a moderate to vigorous aerobic exercising program should be excluded using the recommendations of the ATS (American Thoracic Society) (25), which majorly includes acute and decompensated cardiac, vascular, and pulmonary diseases. Patients with an increased cardiovascular risk are recommended for CPET on an ergometer or any other maximal exercise test. Taking the considerably low serious adverse event rates into account, i.e., 1 out of 10,000 cardiac events and 2–5 deaths out of 100,000 in large cohorts (50), allows for a risk-adapted ergometry testing in high-risk, (borderline) symptomatic patients before assigning them to a prehabilitation program. The exercises can be based on the recommendations of the World Health Organization for physical activity (79). The duration, frequency, and elements of the program can be built around these recommendations and adapted after risk–benefit analysis in an ongoing fashion. Special attention and risk–benefit estimation should be put on the progress of malignancies as every 4 weeks of treatment delay (not only surgical) will increase the risk for tumor progression, which calls for programs that last up to 3 weeks in patients without neoadjuvant strategies and up to 6 weeks in benign or neoadjuvant settings before surgery (46).

Nutrition, patient–blood management, and psychosocial wellbeing have been described as fundamental pillars of prehabilitation. Still, considering this analysis, these items feel like side dishes when compared to the effects mediated by exercise interventions. Screening helps identify patients at increased risk for surgical site infections and other perioperative complications. Perioperative nutrition is critical to fill protein resources, and immunonutrition has been shown to be associated with beneficial and adverse event-reducing effects after surgery (23, 75). PBM as a bundle program can help reduce the number of operations in anemic patients, as anemia is known to be associated with adverse outcomes. It can help reduce the number of transfusions, which are associated with increased mortality (14, 15), and psychosocial wellbeing is important to keep the patients motivated and stay focused before, during, and after the surgical treatment (34, 43, 49, 69). Even if there are contradicting and heterogeneous data on the effect of the above-mentioned items, they might have a granular influence on the complete patient experience, but their effects are hard to measure. In the perception of the authors, they are positive cofounding and surrogate factors for the success and penetration of prehabilitation and should be definitively communicated and implemented in each clinical program. Currently, factors like a stable quality of life and the increase in motivation reflected by high compliance seem to be the clinically best accessible factors.

One aspect that has not been specifically highlighted in the systematic review is smoking cessation. Indeed, this is a critical aspect of improved clinical outcome after surgery and is strongly recommended in the European Code Against Cancer (https://cancer-code-europe.iarc.fr/index.php/en/), and there is a specific World Health Organization Knowledge Summary on key facts about smoking cessation that should be recommended before every surgery that are beneficial for our patients (80).

The economic potential of prehabilitation has not yet been systematically evaluated, but there seems to be a significant return on investment for hospitals that try to get their patients involved in prehabilitation. The data that were analyzed in this systematic and pragmatic review justify an investment into prehabilitation by hospitals as they reduce complications and generate better outcomes for their surgical cohorts. It affects patients that require abdominal operations just as it affects patients with thoracic indications. A lower number of complications usually go hand in hand with less use of intensive care resources, shorter hospital stays, and reduced overall treatment costs. The published data indicate a potential of up to 800% in return on investment (28, 31, 42, 47). An example of an return on investment (ROI) is the availability of hospital days that can be used for additional patients. Staff experience less trauma or frustration due to better outcomes, better quality, and increased safety for the patients (27, 81–83). These are only two among a broad spectrum of factors becoming more and more important in the political demand and obligation toward transparent and risk-adjusted hospital quality reports per indication in most healthcare systems. This is to empower patients to choose the best available treatment location and to shift the payment system to a pay-for-performance approach. Insurances and other stakeholders should establish a reimbursement system for qualified prehabilitation as soon as possible to enable surgeons and anesthesiologists to modify early determinants (physical, nutritional, and psychological state) of late outcomes (morbidity and mortality). This could already start at the referring General Practitioner (GP) level and would foster the collaboration between the ambulatory and hospital sector in a proactive way. The potential of remote digitized solutions should be explored to empower and involve patients and decompress infrastructures on the GP level and at hospitals.

The limitation of this review is its reduced specification, as suggestions are based on the inclusion of all oncologic and major pathologies in the abdomen and thorax. However, patients in these indications are comparable with each other, and the increase in exercise capacity can be regarded as the main goal in improving resistance to postoperative complications. Reasons for excluding other indications were obvious. Cardiosurgical patients often have contraindications against a potentially unsupervised moderate to vigorous aerobic exercise program although the major aim is to increase their functional capacity. Trauma and orthopedic patients, on the other hand, have injuries or physical limitations that indicate a more muscle-strength-focused program. A definite strength of the review is that class 1 and 2 evidence studies were included in the data extraction and evaluation process, indicating high reliability of the published data, except for one exceptional class 3 trial in the economy. Finally, the proposal of a clinical pathway with synergistic and complementary parts shows how prehabilitation might optimally be implemented into the daily hospital and outpatient workflow, including a focused risk management and outcome data measurement to enable penetration and reduce barriers. There are numerous options for further deployment of the tools identified. The authors are currently working on the development of a medical device and have tested it in a pilot study (https://drks.de/search/de/trial/DRKS00026985). A randomized controlled trial is currently set up by the group to explore the potential of remote exercising. In the future, artificial intelligence (AI) applications could analyze the baseline assessments as well as the exercising data and create an AI-based program or directly intervene, tailor, and adapt the individual programs (84).

In conclusion, prehabilitation is a new field in surgery and perioperative medicine requiring definition, assessment, and active quality- and evidence-based approaches, as well as rapid action by stakeholders to establish prehabilitation as a reimbursable instrument for better patient care and increased safety and quality of surgical care. It is not a lifestyle but a critical mosaic stone in a professional and successful surgical treatment strategy with a tremendous economic potential serving for better patient care.

## References

[1] Nepogodiev D Martin J Biccard B Makupe A Bhangu A Nepogodiev D Global burden of postoperative death. Lancet. (2019) 393:401. 10.1016/S0140-6736(18)33139-8 30722955

[2] WHO. WHO guidelines for safe surgery: safe surgery saves lives. WHO (n.d.). Available at: https://www.who.int/patientsafety/safesurgery/tools_resources/9789241598552/en/ (Accessed May 23, 2020).

[3] WHO. 10 facts on patient safety. WHO (n.d.). Available at: http://www.who.int/features/factfiles/patient_safety/en/ (Accessed May 23, 2020).

[4] Shinall MC Arya S Youk A Varley P Shah R Massarweh NN Association of preoperative patient frailty and operative stress with postoperative mortality. JAMA Surg. (2020) 155:e194620. 10.1001/jamasurg.2019.4620 31721994 PMC6865246

[5] Shinall MC Youk A Massarweh NN Shireman PK Arya S George EL Association of preoperative frailty and operative stress with mortality after elective vs emergency surgery. JAMA Netw Open. (2020) 3:e2010358. 10.1001/jamanetworkopen.2020.10358 32658284 PMC7358909

[6] Clavien PA Barkun J de Oliveira ML Vauthey JN Dindo D Schulick RD The Clavien-Dindo classification of surgical complications: five-year experience. Ann Surg. (2009) 250:187–96. 10.1097/SLA.0b013e3181b13ca2 19638912

[7] Dindo D Demartines N Clavien P-A. Classification of surgical complications: a new proposal with evaluation in a cohort of 6336 patients and results of a survey. Ann Surg. (2004) 240:205–13. 10.1097/01.sla.0000133083.54934.ae 15273542 PMC1360123

[8] Slankamenac K Graf R Barkun J Puhan MA Clavien P-A. The comprehensive complication index: a novel continuous scale to measure surgical morbidity. Ann Surg. (2013) 258:1–7. 10.1097/SLA.0b013e318296c732 23728278

[9] Slankamenac K Nederlof N Pessaux P de Jonge J Wijnhoven BPL Breitenstein S The comprehensive complication index: a novel and more sensitive endpoint for assessing outcome and reducing sample size in randomized controlled trials. Ann Surg. (2014) 260:757–62; discussion 762–3. 10.1097/SLA.0000000000000948 25379846

[10] Clavien P-A Vetter D Staiger RD Slankamenac K Mehra T Graf R The comprehensive complication index (CCI®): added value and clinical perspectives 3 years “down the line.” Ann Surg. (2017) 265:1045–50. 10.1097/SLA.0000000000002132 28486288

[11] (n.d.). Available at: https://gco.iarc.fr/.

[12] Haynes AB Weiser TG Berry WR A surgical safety checklist to reduce morbidity and mortality in a global population. N Engl J Med. (2009) 360:491–9. 10.1056/NEJMsa0810119 19144931

[13] Bates DW Levine DM Salmasian H Syrowatka A Shahian DM Lipsitz S The safety of inpatient health care. N Engl J Med. (2023) 388:142–53. 10.1056/NEJMsa2206117 36630622

[14] Meybohm P Fischer DP Geisen C Müller MM Weber CF Herrmann E Safety and effectiveness of a patient blood management (PBM) program in surgical patients—the study design for a multi-centre prospective epidemiologic non-inferiority trial. BMC Health Serv Res. (2014) 14:576. 10.1186/s12913-014-0576-3 25927460 PMC4261241

[15] Meybohm P Herrmann E Steinbicker AU Wittmann M Gruenewald M Fischer D Patient blood management is associated with a substantial reduction of red blood cell utilization and safe for patient’s outcome: a prospective, multicenter cohort study with a noninferiority design. Ann Surg. (2016) 264:203–11. 10.1097/SLA.0000000000001747 27163948

[16] Keding V Zacharowski K Bechstein WO Meybohm P Schnitzbauer AA. Patient blood management improves outcome in oncologic surgery. World J Surg Oncol. (2018) 16:159. 10.1186/s12957-018-1456-9 30086770 PMC6081799

[17] Schnitzbauer AA Eberhard J Bartsch F Brunner SM Ceyhan GO Walter D The MEGNA score and preoperative anemia are major prognostic factors after resection in the German intrahepatic cholangiocarcinoma cohort. Ann Surg Oncol. (2019) 27(4):1147–55. 10.1245/s10434-019-07968-7 31646454

[18] Martin D Besson C Pache B Michel A Geinoz S Gremeaux-Bader V Feasibility of a prehabilitation program before major abdominal surgery: a pilot prospective study. J Int Med Res. (2021) 49:03000605211060196. 10.1177/03000605211060196 34851778 PMC8649915

[19] Liberati A Altman DG Tetzlaff J Mulrow C Gøtzsche PC Ioannidis JPA The PRISMA statement for reporting systematic reviews and meta-analyses of studies that evaluate health care interventions: explanation and elaboration. J Clin Epidemiol. (2009) 62:e1–34. 10.1016/j.jclinepi.2009.06.006 19631507

[20] Howick J Chalmers I Glasziou P Greenhalgh T Heneghan C Liberati A “The 2011 Oxford CEBM Evidence Levels of Evidence (Introductory Document)”. Oxford Centre for Evidence-Based Medicine. Available at: http://www.cebm.net/index.aspx?o=5653. (Accessed February 23, 2023)

[21] Guyatt GH Oxman AD Vist GE Kunz R Falck-Ytter Y Alonso-Coello P GRADE: an emerging consensus on rating quality of evidence and strength of recommendations. Br Med J. (2008) 336:924–6. 10.1136/bmj.39489.470347.AD 18436948 PMC2335261

[22] Current Guidelines | health.gov (n.d.). Available at: https://health.gov/our-work/nutrition-physical-activity/physical-activity-guidelines/current-guidelines (Accessed January 1, 2023).

[23] Arends J Bachmann P Baracos V Barthelemy N Bertz H Bozzetti F ESPEN guidelines on nutrition in cancer patients. Clin Nutr Edinb Scotl. (2017) 36:11–48. 10.1016/j.clnu.2016.07.015 27637832

[24] Arya S Varley P Youk A Borrebach JD Perez S Massarweh NN Recalibration and external validation of the risk analysis index: a surgical frailty assessment tool. Ann Surg. (2020) 272:996–1005. 10.1097/SLA.0000000000003276 30907757 PMC8785437

[25] ATS Committee on Proficiency Standards for Clinical Pulmonary Function Laboratories. ATS statement: guidelines for the six-minute walk test. Am J Respir Crit Care Med. (2002) 166:111–7. 10.1164/ajrccm.166.1.at1102 12091180

[26] Bagaria SP Heckman MG Diehl NN Parker A Wasif N. Delay to colectomy and survival for patients diagnosed with colon cancer. J Investig Surg. (2019) 32:350–7. 10.1080/08941939.2017.1421732 29351008

[27] Barberan-Garcia A Ubré M Roca J Lacy AM Burgos F Risco R Personalised prehabilitation in high-risk patients undergoing elective major abdominal surgery: a randomized blinded controlled trial. Ann Surg. (2018) 267:50–6. 10.1097/SLA.0000000000002293 28489682

[28] Barberan-Garcia A Ubre M Pascual-Argente N Risco R Faner J Balust J Post-discharge impact and cost-consequence analysis of prehabilitation in high-risk patients undergoing major abdominal surgery: secondary results from a randomised controlled trial. Br J Anaesth. (2019) 123:450–6. 10.1016/j.bja.2019.05.032 31248644

[29] Bhatia C Kayser B. Preoperative high-intensity interval training is effective and safe in deconditioned patients with lung cancer: a randomized clinical trial. J Rehabil Med. (2019) 51:712–8. 10.2340/16501977-2592 31468059

[30] Boden I Skinner EH Browning L Reeve J Anderson L Hill C Preoperative physiotherapy for the prevention of respiratory complications after upper abdominal surgery: pragmatic, double blinded, multicentre randomised controlled trial. Br Med J. (2018) 360:j5916. 10.1136/bmj.j5916 29367198 PMC5782401

[31] Boden I Robertson IK Neil A Reeve J Palmer AJ Skinner EH Preoperative physiotherapy is cost-effective for preventing pulmonary complications after major abdominal surgery: a health economic analysis of a multicentre randomised trial. J Physiother. (2020) 66:180–7. 10.1016/j.jphys.2020.06.005 32680742

[32] Bolshinsky V Li MH-G Ismail H Burbury K Riedel B Heriot A. Multimodal prehabilitation programs as a bundle of care in gastrointestinal cancer surgery: a systematic review. Dis Colon Rectum. (2018) 61:124–38. 10.1097/DCR.0000000000000987 29219922

[33] Bourgade V Drouin SJ Yates DR Parra J Bitker M-O Cussenot O Impact of the length of time between diagnosis and surgical removal of urologic neoplasms on survival. World J Urol. (2014) 32:475–9. 10.1007/s00345-013-1045-z 23455886

[34] Briggs LG Reitblat C Bain PA Parke S Lam N-Y Wright J Prehabilitation exercise before urologic cancer surgery: a systematic and interdisciplinary review. Eur Urol. (2022) 81:157–67. 10.1016/j.eururo.2021.05.015 34074558

[35] Haynes AB Weiser TG Berry WR Lipsitz SR Breizat A-HS Dellinger EP Oral nutrition as a form of pre-operative enhancement in patients undergoing surgery for colorectal cancer: a systematic review. Surg Infect. (2018) 19:1–10. 10.1089/sur.2017.143 29049000

[36] Cavalheri V Granger C. Preoperative exercise training for patients with non-small cell lung cancer. Cochrane Database Syst Rev. (2017) 6:CD012020. 10.1002/14651858.CD012020.pub2 28589547 PMC6481477

[37] Ekblom-Bak E Ekblom B Söderling J Börjesson M Blom V Kallings LV Sex- and age-specific associations between cardiorespiratory fitness, CVD morbidity and all-cause mortality in 266.109 adults. Prev Med. (2019) 127:105799. 10.1016/j.ypmed.2019.105799 31454664

[38] Ekblom-Bak E Stenling A Salier Eriksson J Hemmingsson E Kallings LV Andersson G Latent profile analysis patterns of exercise, sitting and fitness in adults—associations with metabolic risk factors, perceived health, and perceived symptoms. PLoS One. (2020) 15:e0232210. 10.1371/journal.pone.0232210 32330191 PMC7182226

[39] Ekblom-Bak E Halldin M Vikström M Stenling A Gigante B de Faire U Physical activity attenuates cardiovascular risk and mortality in men and women with and without the metabolic syndrome—a 20-year follow-up of a population-based cohort of 60-year-olds. Eur J Prev Cardiol. (2021) 28:1376–85. 10.1177/2047487320916596 34647588

[40] Elit LM O’Leary EM Pond GR Seow H-Y. Impact of wait times on survival for women with uterine cancer. J Clin Oncol. (2013) 32(1):27–33. 10.1200/JCO.2013.51.3671 24276779

[41] Figueiredo N Panteleimonitis S Popeskou S Cunha JF Qureshi T Beets GL Delaying surgery after neoadjuvant chemoradiotherapy in rectal cancer has no influence in surgical approach or short-term clinical outcomes. Eur J Surg Oncol. (2018) 44:484–9. 10.1016/j.ejso.2018.01.088 29398323

[42] Fuertes-Guiró F Viteri Velasco E. The impact of frailty on the economic evaluation of geriatric surgery: hospital costs and opportunity costs based on meta-analysis. J Med Econ. (2020) 23(8):819–30. 10.1080/13696998.2020.1764965 32372679

[43] Fulop A Lakatos L Susztak N Szijarto A Banky B. The effect of trimodal prehabilitation on the physical and psychological health of patients undergoing colorectal surgery: a randomised clinical trial. Anaesthesia. (2021) 76:82–90. 10.1111/anae.15215 32761611

[44] Giannitsi S Bougiakli M Bechlioulis A Kotsia A Michalis LK Naka KK. 6-minute walking test: a useful tool in the management of heart failure patients. Ther Adv Cardiovasc Dis. (2019) 13:1753944719870084. 10.1177/1753944719870084 31441375 PMC6710700

[45] Hall DE Arya S Schmid KK Blaser C Carlson MA Bailey TL Development and initial validation of the risk analysis index for measuring frailty in surgical populations. JAMA Surg. (2017) 152:175–82. 10.1001/jamasurg.2016.4202 27893030 PMC7140150

[46] Hanna TP King WD Thibodeau S Jalink M Paulin GA Harvey-Jones E Mortality due to cancer treatment delay: systematic review and meta-analysis. Br Med J. (2020) 371:m4087. 10.1136/bmj.m4087 33148535 PMC7610021

[47] Howard R Yin YS McCandless L Wang S Englesbe M Machado-Aranda D. Taking control of your surgery: impact of a prehabilitation program on major abdominal surgery. J Am Coll Surg. (2019) 228:72–80. 10.1016/j.jamcollsurg.2018.09.018 30359831 PMC6309718

[48] Lambert JE Hayes LD Keegan TJ Subar DA Gaffney CJ. The impact of prehabilitation on patient outcomes in hepatobiliary, colorectal, and upper gastrointestinal cancer surgery: a PRISMA-accordant meta-analysis. Ann Surg. (2021) 274:70–7. 10.1097/SLA.0000000000004527 33201129

[49] Lau CSM Chamberlain RS. Prehabilitation programs improve exercise capacity before and after surgery in gastrointestinal cancer surgery patients: a meta-analysis. J Gastrointest Surg. (2020) 24:2829–37. 10.1007/s11605-019-04436-1 31768827

[50] Levett DZH Jack S Swart M Carlisle J Wilson J Snowden C Perioperative cardiopulmonary exercise testing (CPET): consensus clinical guidelines on indications, organization, conduct, and physiological interpretation. Br J Anaesth. (2018) 120:484–500. 10.1016/j.bja.2017.10.020 29452805

[51] Liu C Lu Z Zhu M Lu X. Trimodal prehabilitation for older surgical patients: a systematic review and meta-analysis. Aging Clin Exp Res. (2022) 34:485–94. 10.1007/s40520-021-01929-5 34227052

[52] Looijaard SMLM Slee-Valentijn MS Otten RHJ Maier AB. Physical and nutritional prehabilitation in older patients with colorectal carcinoma: a systematic review. J Geriatr Phys Ther. (2018) 41:236–44. 10.1519/JPT.0000000000000125 28252474

[53] McKenna NP Bews KA Al-Refaie WB Colibaseanu DT Pemberton JH Cima RR Assessing malnutrition before major oncologic surgery: one size does not fit all. J Am Coll Surg. (2020) 230:451–60. 10.1016/j.jamcollsurg.2019.12.034 32113029

[54] Meng X Press B Renson A Wysock JS Taneja SS Huang WC Discriminative ability of commonly used indexes to predict adverse outcomes after radical cystectomy: comparison of demographic data, American Society of Anesthesiologists, Modified Charlson Comorbidity Index, and Modified Frailty Index. Clin Genitourin Cancer. (2018) 16:e843–50. 10.1016/j.clgc.2018.02.009 29550199

[55] Minnella EM Awasthi R Gillis C Fiore JF Liberman AS Charlebois P Patients with poor baseline walking capacity are most likely to improve their functional status with multimodal prehabilitation. Surgery. (2016) 160:1070–9. 10.1016/j.surg.2016.05.036 27476586

[56] Minnella EM Awasthi R Bousquet-Dion G Ferreira V Austin B Audi C Multimodal prehabilitation to enhance functional capacity following radical cystectomy: a randomized controlled trial. Eur Urol Focus. (2021) 7:132–8. 10.1016/j.euf.2019.05.016 31186173

[57] Mirkin KA Hollenbeak CS Wong J. Time to surgery: a misguided quality metric in early stage pancreatic cancer. J Gastrointest Surg. (2018) 22:1365–75. 10.1007/s11605-018-3730-0 29520648

[58] Moran J Guinan E McCormick P Larkin J Mockler D Hussey J The ability of prehabilitation to influence postoperative outcome after intra-abdominal operation: a systematic review and meta-analysis. Surgery. (2016) 160:1189–201. 10.1016/j.surg.2016.05.014 27397681

[59] Palumbo C Knipper S Pecoraro A Rosiello G Luzzago S Deuker M Patient frailty predicts worse perioperative outcomes and higher cost after radical cystectomy. Surg Oncol. (2020) 32:8–13. 10.1016/j.suronc.2019.10.014 31683158

[60] Piraux E Reychler G de Noordhout LM Forget P Deswysen Y Caty G. What are the impact and the optimal design of a physical prehabilitation program in patients with esophagogastric cancer awaiting surgery? A systematic review. BMC Sports Sci Med Rehabil. (2021) 13:33. 10.1186/s13102-021-00260-w 33766107 PMC7993458

[61] Polverini AC Nelson RA Marcinkowski E Jones VC Lai L Mortimer JE Time to treatment: measuring quality breast cancer care. Ann Surg Oncol. (2016) 23:3392–402. 10.1245/s10434-016-5486-7 27503492

[62] Probst P Ohmann S Klaiber U Hüttner FJ Billeter AT Ulrich A Meta-analysis of immunonutrition in major abdominal surgery. Br J Surg. (2017) 104:1594–608. 10.1002/bjs.10659 28940219

[63] Shah R Attwood K Arya S Hall DE Johanning JM Gabriel E Association of frailty with failure to rescue after low-risk and high-risk inpatient surgery. JAMA Surg. (2018) 153:e180214. 10.1001/jamasurg.2018.0214 29562073 PMC5875343

[64] Shah R Borrebach JD Hodges JC Varley PR Wisniewski MK Shinall MC Validation of the risk analysis index for evaluating frailty in ambulatory patients. J Am Geriatr Soc. (2020) 68:1818–24. 10.1111/jgs.16453 32310317 PMC7725401

[65] Sheill G Reynolds S O’Neill L Mockler D Reynolds JV Hussey J Cardiopulmonary exercise testing in oesophagogastric surgery: a systematic review. J Gastrointest Surg. (2020) 24:2667–78. 10.1007/s11605-020-04696-2 32632727

[66] Simunovic M Rempel E Thériault M-E Baxter NN Virnig BA Meropol NJ Influence of delays to nonemergent colon cancer surgery on operative mortality, disease-specific survival and overall survival. Can J Surg J Can Chir. (2009) 52:E79–86. PMID: ; PMCID: 19680502 PMC2724831

[67] Strohl AE Feinglass JM Shahabi S Simon MA. Surgical wait time: a new health indicator in women with endometrial cancer. Gynecol Oncol. (2016) 141:511–5. 10.1016/j.ygyno.2016.04.014 27103178 PMC5989709

[68] Tew GA Ayyash R Durrand J Danjoux GR. Clinical guideline and recommendations on pre-operative exercise training in patients awaiting major non-cardiac surgery. Anaesthesia. (2018) 73:750–68. 10.1111/anae.14177 29330843

[69] Thillainadesan J Yumol MF Hilmer S Aitken SJ Naganathan V. Interventions to improve clinical outcomes in older adults admitted to a surgical service: a systematic review and meta-analysis. J Am Med Dir Assoc. (2020) 21:1833–43.e20. 10.1016/j.jamda.2020.03.023 32417101

[70] van Kooten RT Bahadoer RR Peeters KCMJ Hoeksema JHL Steyerberg EW Hartgrink HH Preoperative risk factors for major postoperative complications after complex gastrointestinal cancer surgery: a systematic review. Eur J Surg Oncol. (2021) 47:3049–58. 10.1016/j.ejso.2021.07.021 34340874

[71] Varley PR Borrebach JD Arya S Massarweh NN Bilderback AL Wisniewski MK Clinical utility of the risk analysis index as a prospective frailty screening tool within a multi-practice, multi-hospital integrated healthcare system. Ann Surg. (2021) 274:e1230–7. 10.1097/SLA.0000000000003808 32118596

[72] Waterland JL McCourt O Edbrooke L Grangessr CL Ismail H Riedel B Efficacy of prehabilitation including exercise on postoperative outcomes following abdominal cancer surgery: a systematic review and meta-analysis. Front Surg. (2021) 8:628848. 10.3389/fsurg.2021.628848 33816546 PMC8017317

[73] Weimann A Breitenstein S Breuer JP Gabor SE Holland-Cunz S Kemen M Clinical nutrition in surgery. Guidelines of the German Society for Nutritional Medicine. Chir Z Alle Geb Oper Medizen. (2014) 85:320–6. 10.1007/s00104-014-2737-7 24718444

[74] Xu K Watanabe-Galloway S Rochling FA Farazi PA Monirul Islam KM Wang H Surgical delay is associated with improved survival in hepatocellular carcinoma: results of the national cancer database. J Gastrointest Surg. (2019) 23:933–43. 10.1007/s11605-018-3925-4 30328070 PMC12110860

[75] Yu K Zheng X Wang G Liu M Li Y Yu P Immunonutrition vs standard nutrition for cancer patients: a systematic review and meta-analysis (part 1). JPEN J Parenter Enteral Nutr. (2020) 44:742–67. 10.1002/jpen.1736 31709584

[76] Richards T Baikady RR Clevenger B Butcher A Abeysiri S Chau M Preoperative intravenous iron to treat anaemia before major abdominal surgery (PREVENTT): a randomised, double-blind, controlled trial. Lancet. (2020) 396:1353–61. 10.1016/S0140-6736(20)31539-7 32896294 PMC7581899

[77] Faqar-Uz-Zaman SF Filmann N Mahkovic D von Wagner M Detemble C Kippke U Study protocol for a prospective, double-blinded, observational study investigating the diagnostic accuracy of an app-based diagnostic health care application in an emergency room setting: the eRadaR trial. BMJ Open. (2021) 11:e041396. 10.1136/bmjopen-2020-041396 33419909 PMC7798704

[78] Faqar-Uz-Zaman SF Anantharajah L Baumartz P Sobotta P Filmann N Zmuc D The diagnostic efficacy of an app-based diagnostic health care application in the emergency room: eRadaR-trial. A prospective, double-blinded, observational study. Ann Surg. (2022) 276:935–42. 10.1097/SLA.0000000000005614 35925755

[79] Physical activity (n.d.). Available at: https://www.who.int/news-room/fact-sheets/detail/physical-activity (Accessed January 22, 2023).

[80] Yoong SL Tursan d’Espaignet E Wiggers J St Claire S Mellin-Olsen J Grady A Tobacco and postsurgical outcomes: WHO tobacco knowledge summaries. Geneva: World Health Organization (2020). Available at: https://www.google.com/search?client=firefox-b-d&q=Yoong+SL%2C+Tursan+d%E2%80%99Espaignet+E%2C+Wiggers+J%2C+St+Claire+S%2C+Mellin-Olsen+J%2C+Grady+A+et+al.+Tobacco+and+postsurgical+outcomes%3A+WHO+tobacco+knowledge+summaries.+Geneva%3A+World+Health+Organization%3B+2020.+Licence%3A+CC+BY-NC-SA+3.0+IGO (Accessed April 5, 2023).

[81] Joliat G-R Demartines N Uldry E. Systematic review of the impact of patient death on surgeons. Br J Surg. (2019) 106:1429–32. 10.1002/bjs.11264 31373690

[82] Bohnen JD Lillemoe KD Mort EA Kaafarani HMA. When things go wrong: the surgeon as second victim. Ann Surg. (2019) 269:808–9. 10.1097/SLA.0000000000003138 30480564

[83] Han K Bohnen JD Peponis T Martinez M Nandan A Yeh DD The surgeon as the second victim? Results of the Boston intraoperative adverse events surgeons’ attitude (BISA) study. J Am Coll Surg. (2017) 224:1048–56. 10.1016/j.jamcollsurg.2016.12.039 28093300

[84] Taher H Grasso V Tawfik S Gumbs A. The challenges of deep learning in artificial intelligence and autonomous actions in surgery: a literature review. Artif Intell Surg. (2022) 2:144–58. 10.20517/ais.2022.11

